# HEARING LOSS AND SPOUSE’S MENTAL HEALTH WITH GENDER DIFFERENCES

**DOI:** 10.1093/geroni/igae098.3433

**Published:** 2024-12-31

**Authors:** Jaemin Jeon, Kyuho Lee

**Affiliations:** Daegu University, Gyeongsan, Kyongsang-bukto, Republic of Korea; Daegu University, Gyeongsan, Kyongsang-bukto, Republic of Korea

## Abstract

This research investigated the influence of individuals’ auditory acuity on the occurrence of depressive symptoms in their spouses, mediated by the provision of support within marital relationships among elderly couples. Data from a sample of 1,069 older adult couples participating in the Health and Retirement Study (HRS) were analyzed. Utilizing three-wave dyadic data collected in 2010, 2014, and 2018, we conducted path analyses employing Mplus 7.0 to estimate the mediating effects of spousal support while controlling for autoregressive effects. Our findings indicate that husbands’ superior auditory acuity at the initial assessment (T1) was associated with a reduction in depressive symptoms among their wives at the third assessment point (T3). Spousal support served as a mediator in this relationship. However, wives’ auditory acuity did not exhibit a longitudinal impact on their husbands’ depressive symptoms. This study provides empirical evidence supporting the significant role of older adults’ auditory abilities within the context of marital relationships and the mental well-being of spouses. Keywords: hearing, spousal support, depressive symptoms

